# The Making of Long-Lasting Memories: A Fruit Fly Perspective

**DOI:** 10.3389/fnbeh.2021.662129

**Published:** 2021-03-30

**Authors:** Camilla Roselli, Mani Ramaswami, Tamara Boto, Isaac Cervantes-Sandoval

**Affiliations:** ^1^Trinity College Institute of Neuroscience, School of Genetics and Microbiology, Smurfit Institute of Genetics and School of Natural Sciences, Trinity College Dublin, Dublin, Ireland; ^2^National Centre for Biological Sciences, TIFR, Bengaluru, India; ^3^Trinity College Institute of Neuroscience, Department of Physiology, School of Medicine, Trinity College Dublin, Dublin, Ireland; ^4^Department of Biology, Georgetown University, Washington, DC, United States; ^5^Interdisciplinary Program in Neuroscience, Georgetown University, Washington, DC, United States

**Keywords:** long-term memories, synaptic plasiticity, protein synthesis, prion-like protein, behavioral neuroscience

## Abstract

Understanding the nature of the molecular mechanisms underlying memory formation, consolidation, and forgetting are some of the fascinating questions in modern neuroscience. The encoding, stabilization and elimination of memories, rely on the structural reorganization of synapses. These changes will enable the facilitation or depression of neural activity in response to the acquisition of new information. In other words, these changes affect the weight of specific nodes within a neural network. We know that these plastic reorganizations require *de novo* protein synthesis in the context of Long-term memory (LTM). This process depends on neural activity triggered by the learned experience. The use of model organisms like *Drosophila melanogaster* has been proven essential for advancing our knowledge in the field of neuroscience. Flies offer an optimal combination of a more straightforward nervous system, composed of a limited number of cells, and while still displaying complex behaviors. Studies in *Drosophila* neuroscience, which expanded over several decades, have been critical for understanding the cellular and molecular mechanisms leading to the synaptic and behavioral plasticity occurring in the context of learning and memory. This is possible thanks to sophisticated technical approaches that enable precise control of gene expression in the fruit fly as well as neural manipulation, like chemogenetics, thermogenetics, or optogenetics. The search for the identity of genes expressed as a result of memory acquisition has been an active interest since the origins of behavioral genetics. From screenings of more or less specific candidates to broader studies based on transcriptome analysis, our understanding of the genetic control behind LTM has expanded exponentially in the past years. Here we review recent literature regarding how the formation of memories induces a rapid, extensive and, in many cases, transient wave of transcriptional activity. After a consolidation period, transcriptome changes seem more stable and likely represent the synthesis of new proteins. The complexity of the circuitry involved in memory formation and consolidation is such that there are localized changes in neural activity, both regarding temporal dynamics and the nature of neurons and subcellular locations affected, hence inducing specific temporal and localized changes in protein expression. Different types of neurons are recruited at different times into memory traces. In LTM, the synthesis of new proteins is required in specific subsets of cells. This *de novo* translation can take place in the somatic cytoplasm and/or locally in distinct zones of compartmentalized synaptic activity, depending on the nature of the proteins and the plasticity-inducing processes that occur. We will also review recent advances in understanding how localized changes are confined to the relevant synapse. These recent studies have led to exciting discoveries regarding proteins that were not previously involved in learning and memory processes. This invaluable information will lead to future functional studies on the roles that hundreds of new molecular actors play in modulating neural activity.

## Introduction

Long-term memory (LTM), which is stable for days, months, years, or a lifetime, is classically distinguished from transient, short-term memory (STM) by the need for altered nuclear gene expression and *de novo* protein synthesis. Short-term memories are translation-independent, form rapidly, and last for short periods of time, seconds to hours (Stough et al., 2006). In contrast, LTM formation requires initial acquisition through specific experiences or training protocols, during which the organism forms a labile form of memory that can be disrupted by seizure, new learning, or inhibition of specific cellular processes (Izquierdo et al., 2002). LTM formation also requires a subsequent slow, protein-synthesis-dependent consolidation process that makes the newly formed memory resistant to disruption (Bailey et al., 1996). LTM is maintained, i.e., the mechanisms through which memories outlast the molecular turnover to maintain themselves remain unclear, but nuclear, cytosolic, and synaptic mechanisms have all been invoked (Sudhakaran and Ramaswami, 2017).

Additional observations have modified and enhanced this classical view. In particular, by showing that: (a) consolidation involves not only cellular mechanisms but also systems-level mechanisms in which memories become more widely distributed across the brain; and (b) during retrieval, long-term memories become subject to updating, modification, or reconsolidation (Lee, 2008). Moreover, new work has informed molecular and systems mechanisms of memory recall, extinction, and forgetting.

As previously mentioned, LTM requires the production of new proteins in organisms ranging from sea slugs to mice. This was generally shown by observing the effect of protein synthesis inhibitors (PSI) on different phases of memory. Intracranial injection of PSI into the goldfish after training session, blocks the formation of long-term, but not short-term shock avoidance memory (Agranoff et al., 1967). Similarly, injecting mice with PSI 30 min before training results in 24 h amnesia; interestingly, protein synthesis inhibitor injection after training has no effect on 24-h memory (Squire and Barondes, 1972), suggesting the requirement of translational activation during a specific temporal window. In invertebrates, fruit flies fed with PSI before a spaced-training protocol, which usually results in long-lasting (24–96 h) memory, show normal early memory but disrupted LTM (Tully et al., 1994). These experiments suggest the requirement of protein synthesis, specifically during the consolidation phase of memory formation.

In addition, a role for translational control mechanisms for LTM has been revealed in *Drosophila* through the analysis of a number of genetic mutants. Specifically, mutations in several translational control components, such as: *pumilio*, *staufen*, *eIF-5C* (Dubnau et al., 2003), polyA polymerase *gld2* (Kwak et al., 2008), *dfmr1*, and elements of the miRNA pathway (Bolduc et al., 2008; Sudhakaran et al., 2014), have been found to selectively impair long-term but not STM.

Despite remarkable progress in the understanding of protein synthesis dependent-LTM, some questions remain open: what is the molecular nature of engram, in other words, what is the identity and the mechanisms of proteins involved in the formation and maintenance of LTM? Which translational mechanisms are specifically engaged in LTM formation? How are these mechanisms constrained to relevant synapses? In which neuronal types and at which time of memory formation are these translational mechanisms required? Moreover, in certain circumstances, long-lasting memories can be formed in the absence of protein synthesis; how are these memories formed, and how are they maintained? Here we review recent literature that continues to shed some light on these critical questions.

## Transcriptional Modulation by Long-Term Memory Formation

Behavioral flexibility is essential for survival, and it relies on the plasticity of the nervous system. This is structurally maintained by reorganization of synapses and mobilization of cellular components. For the last decades, much work has been put into understanding how neurons that cannot rely on cell division maintain their adaptative plasticity as postmitotic cells and how those changes are stabilized with time (Bailey et al., 1994; Okamoto et al., 2004; Neves et al., 2008; Ho et al., 2011; Zhao et al., 2011; Frank, 2014; Sugie et al., 2015). Neural activity is known to induce changes in gene expression; even specific early activity genes like *c-fos* are used reliably as markers of neural activity in vertebrates (Dragunow and Faull, 1989).

Neurons couple synaptic activity with the nuclear program to induce new transcript and protein synthesis in order to maintain the physical substrates that allow for long-term storage of new information. STMs require transient changes in the synaptic machinery, but, although different mechanisms of protein synthesis-independent consolidated memory have been described (Zhao et al., 2019; Eschment et al., 2020), the majority of long-lasting memories require new protein synthesis to sustain the generation of enduring and sometimes permanent synaptic modifications (Yin et al., 1994). As mentioned above, the requirement of protein translation in memory consolidation processes becomes evident when using pharmacological approaches to inhibit protein synthesis (Schacher et al., 1988; Tully et al., 1994). Early work in *Aplysia* identified the cAMP response element-binding protein, CREB (Dash et al., 1990), as one of the transcription factors responsible for the recruitment of translational machinery and new protein expression underlying LTM formation and consolidation. Interfering with CREB function affects not only LTM (Yin et al., 1994; Widmer et al., 2018b), but also functional synaptic plasticity (Davis et al., 1996); further confirming that CREB-dependent transcriptional activity is a requisite for the maintenance of the reorganization and persistence of cellular and synaptic alterations that support LTM.

Over the last decades, exhaustive work has been dedicated to identifying the specific nature of the genes involved in memory maintenance. Behavioral mutagenesis screenings in *Drosophila* were initially successful in identifying “classic” memory genes, as well as approaches to reveal CREB downstream targets. These initial screenings identified around ten “learning and memory” genes involved in STM. One of the firsts attempts to elucidate the identity of genes required for LTM formation used a transcriptomics approach based on DNA microarrays to identify differentially expressed genes after spaced aversive olfactory conditioning (Dubnau et al., 2003), where flies are trained to associate an odor with an electric shock. This transcriptomics approach was followed by a behavioral screen, using transposon generated mutations and identified 24 genes which mutants were defective in the expression of 24 h memory (Dubnau et al., 2003). Table 1 presents several genes that have been reported to be differentially expressed after the formation of LTM and confirmed to be functionally involved in memory processes.

**TABLE 1:** Genes that have been reported to be differentially expressed after the formation of LTM and are functionally involved in memory processes.

	**LTM Memory**	**Method**	**Gene/s**
Dubnau et al., 2003	Olfactory aversive conditioning	DNA microarray	*pumilio*/*staufen*
Crocker et al., 2016	Olfactory aversive conditioning	RNAseq of harvested neurons	*pinta*, *Rh4*
Bozler et al., 2017	Wasp-exposure induced memory (non-associative)	RNAseq of whole heads	*IM18*, *a-Try* (associated to adult MB)
Jones et al., 2018	Courtship memory	RNAseq of MB nuclei (*INTACT*)	Previously memory-associated genes: *Orb2*, *staufen*, *oamb*, *Gaq*, and *PKA-R2*
Widmer et al., 2018a	Olfactory appetitive conditioning	Targeted DamID (*TaDa*) in MB	*vajk-1*, *hacd1*, *CG12338*, *mir-282*, and *Cpr64Aa*
Petruccelli et al., 2020	Odor-cue-induced ethanol memory	RNAseq of MB nuclei (*INTACT*)	cdc5*

*spliceosome complex gene cdc5 showcasing the differential expression of alternatively spliced variants.

The development of sophisticated molecular techniques leads to new approaches aiming to investigate the genetic basis of LTM. More recently, differential gene expression has been reported in various neuron types for different memory paradigms. RNA sequencing of whole *Drosophila* heads after the formation of long-term memories using a wasp exposition assay revealed different waves of gene expression (Bozler et al., 2017). When female flies are exposed to predator wasps, they prefer to lay eggs in ethanol containing substrates. This behavior is reported to persist even in the absence of wasps and can be observed several days after the initial exposure. Using this paradigm, the authors found approximately 180 differentially expressed genes (>2-fold change), many of which were previously unknown memory genes, and they were classified into six functional clusters. Interestingly, signal peptides and proteases clusters were highly enriched and knockdown of some of these genes confirmed their involvement in LTM (Bozler et al., 2017).

Similar results were observed using courtship conditioning. During this paradigm, male flies display a learnt behavior by reducing their courtship based on previous exposition to unreceptive females. An initial increment in gene expression is also observed after this conditioning, both in whole heads and in the mushroom body (MB), a structure essential for learning and memory in the fly (Jones et al., 2018). There is a general initial upregulation of genes involved in sensory responses, as well as a transient increase in genes of memory-related pathways in the MB. Interestingly, a down-regulation of genes involved in metabolic function was also observed. Later waves of gene expression include genes deemed essential for the structural and functional changes supporting LTM, including Orb2 (Jones et al., 2018).

Transcriptomic analysis in mnemonic intrinsic and extrinsic MB cells revealed an unknown role of light-sensing proteins in aversive olfactory LTM formation (Crocker et al., 2016). In addition, the authors reported a list of 21 genes that are differentially expressed after spaced conditioning that were previously implicated in memory. In a similar approach, several genes were found to be differentially expressed after the formation of appetitive LTM, and ten were confirmed to have a significant behavioral impact (Widmer et al., 2018a). Not much overlap appears to exist between the results of all different attempts to identify genes behind LTM formation and consolidation. The different findings of the aforementioned studies could be explained taking into consideration the nature of the memories formed (non-associative vs associative, aversive vs appetitive, etc.), the different requirements of the memory inducing paradigms, or ultimately to the specific transcriptomic analysis and experimental design (drivers used to identify cell types, sampling time points, etc.).

Interestingly, in a recent study, transcriptomic analysis of MB nuclei revealed that even though the overall expression of specific genes might remain unchanged, there is differential regulation of the expression of certain transcripts in the context of the formation of alcohol-induced LTM (Petruccelli et al., 2020), where animals are trained to associate an odor with alcohol as unconditioned stimulus. These results emphasize the remarkably refined regulation of the transcriptome profile, with the requirement of different spliced versions of the same gene depending on the memory context.

The aforementioned studies highlight the importance of gene expression and transcriptomic analysis in different contexts and different cell types to eventually identify genes and molecular pathways that make memories last in time. The development of more sophisticated sequencing techniques allowing single-cell transcriptomics of whole brains, have already revealed valuable information on gene expression in the brain and mnemonic cells of the fruit fly (Croset et al., 2018; Davie et al., 2018; Shih et al., 2019), that can lead to high throughput studies of the genetic bases of memory consolidation and maintenance. Many of these approaches mainly target changes in levels of mRNAs present in the nuclei/somatic compartments of neurons. Taking into consideration the intricated morphology associated with neural networks, these analysis could be underestimating events localized in specific projections far away from the neural soma. There is still a long way to go to understand the specific requirements for transcription and new protein synthesis in LTM processes.

## Circuit Mechanisms for the Formation of LTM

**FIGURE 1:**
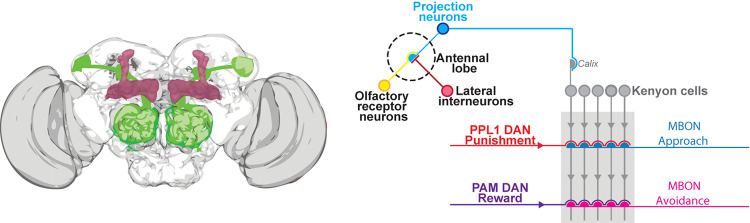
Simplified schematic of the olfactory memory circuit. Left. *Drosophila* brain schematic showing the PN in green and MB lobes in red. Right: the olfactory stimulus is initially detected by one (or multiple) olfactory receptor neurons (ORN) depending on the odorant. The signal is then transmitted to the PN at antennal lobe level. The ORN-PN synapse is modulated by a local interneuron, which is often inhibitory. The PN then synapse onto the KC in a neuropil region called calyx. All together KC axons form a macro-structure called mushroom bodies (MB), which are considered the main center for associative memory. Two different neuronal types establish connections with the MB: Dopaminergic neurons (DAN) and the MB Output Neurons (MBON). DAN drive plasticity onto the KC and are divided into two classes: the paired posterior lateral 1(PPL1) cluster, which encodes punishment, and the protocerebral anterior medial (PAM) cluster, which encodes reward. The MBON mediate the behavioral response which can be either approach or avoidance. During memory encoding, either the aversive or appetitive circuits are activated. In a simplified model, if the learned stimulus is aversive, PPL1-punishment neurons are activated causing plasticity at the MB-MBON (blue) synapse. Plasticity reduces the activity of the blue-MBON which mediate for approach; while the MBON that mediate avoidance (pink), is activated at the same intensity, causing an avoidance behavior. On the other hand, if the learned stimulus is appetitive PAM-reward neurons are activated causing plasticity onto the MB-MBON (pink) synapse. Plasticity reduces the activity of pink-MBON, which mediate avoidance; while the MBON, that mediate approach (blue), is activated at the same intensity, causing an approach behavior. In the interest of simplicity, other important neurons contributing to the MB circuit are excluded.

One initial approach to understand how and where plasticity is induced within a specific neural circuit is to identify the cell types where new protein synthesis is required for proper LTM expression. In *Drosophila*, the initial obvious candidate are the MB cells. The *Drosophila* MB is believed to be the coding center of olfactory memories (Heisenberg et al., 1985; Dubnau and Tully, 2001; McGuire et al., 2001; Siegenthaler et al., 2019). The major sensory input to Kenyon cells (KC), occur in the MB main calyx, where their dendrites receive input from around 180 olfactory projection neurons (PN, Figure 1). Olfactory representation in the KCs is highly sparse across all α/β, α′/β′ and γ KCs (Honegger et al., 2011). This sparseness is generated in part by a negative feedback loop between KCs and the GABAergic anterior-paired lateral neuron (APL) (Lin et al., 2014). During associative memory acquisition, positive or negative values are assigned by associated reward and punishment, respectively. This reinforcement is achieved by the coincident activation of the sparse number KC by odorant and dopaminergic neurons (DAN) that innervate discrete zones, composed by 15 tile-like compartments of the KC lobes. Each of these tiles has corresponding mushroom bodies output neurons (MBON), activation of which favors either approach or avoidance behavior (Aso et al., 2014b; Owald and Waddell, 2015; Berry et al., 2018). The molecular detection of the coincidence that occurs within KCs during learning is thought to change the output weight of KC synapses onto the corresponding MBON, suggesting a model in which dopamine-induced plasticity tilts the overall MBON network to direct appropriate behavior (Owald and Waddell, 2015; Owald et al., 2015). In fact, recent studies have shown that learning alters odor drive to specific MBONs (Séjourné et al., 2011; Pai et al., 2013; Plaçais et al., 2013; Bouzaiane et al., 2015; Hige et al., 2015; Owald et al., 2015; Berry et al., 2018; Cervantes-sandoval et al., 2020). Interestingly, reward learning appears to reduce drive to output pathways that direct avoidance, whereas aversive learning increases drive to avoidance pathways while reducing drive to approach pathways (Owald et al., 2015; Figure 1). Since MBON dendrites cover the same 15 domains tiling pattern of innervation to the lobes as the DAN, it is predictable that they modify the weight of the corresponding MBON exclusively. In fact, it seems that dopaminergic inputs to the KC modulate synaptic transmission with precise spatial specificity, allowing individual KCs to differentially convey olfactory signals to each of their postsynaptic MBON (Boto et al., 2014; Cohn et al., 2015). Individualized modulation of calcium dynamics in specific synaptic boutons in KC after learning highlights the importance of precise functional modifications in specific synapses along the same axon (Bilz et al., 2020), which implies the existence of localized molecular reorganizations. These changes could rely on localized protein expression in LTM, which will be discussed later in this review. Notwithstanding, the DAN > KC > MBON circuit that drives behavior appears to be more complex than this. The existence of putative feedback/feedforward loops (Aso et al., 2014a; Cervantes-Sandoval et al., 2017; Li et al., 2020) suggests the presence of a multi-layered network that regulates the information flow at different levels. It was exquisitely shown that the effect of plasticity in specific KC > MBON-γ1pedc>α/β is not confined to that compartment but by a feedforward inhibition loop it causes changes in the flow of information in MBONβ′2mp and MBONγ5β′2a as well (Perisse et al., 2016). In addition to this complexity, distinct DAN driving synaptic changes between KC and MBON follow different rules (Hige et al., 2015; Aso and Rubin, 2016). This means that different compartments have different plasticity properties. In the context of LTM, spaced pseudo-conditioning using optogenetic activation of ppl1-α′2α2 and γ2α′1 can generate aversive LTM that lasts up to 4 days. In contrast, optogenetic activation of ppl1-γ1 pedc DAN ppl1generates aversive LTM that lasts only 24 h. On the other hand, a single training trial using optogenetic activation of PAM β1, β2, and PAM α1 can generate appetitive LTM (Aso and Rubin, 2016). For the expression of the memory, it has been shown that MBON α2Sc output is required for aversive LTM retrieval (Séjourné et al., 2011), and MBON α3 for appetitive LTM (Plaçais et al., 2013). These findings hold some resonance with foundational findings suggesting that different MB lobes are required for the expression of different phases of memory; for example, initial findings indicated that the vertical lobes of MB were necessary for the formation of LTM (Pascual and Preat, 2001; Yu et al., 2006).

## Spatial Requirement of *de novo* Gene Expression and Protein Synthesis for LTM

The link between neural activity during LTM formation and the activation of a nuclear transcriptional program in KC was revealed by RNA sequencing studies reporting differential gene expression in MB (Jones et al., 2018; Widmer et al., 2018a; Petruccelli et al., 2020) and by the analysis of epigenetic rearrangements associated with later memory phases in the nuclei of the KC (Kramer et al., 2011). The transcriptional machinery required during LTM formation is different than the one required for its maintenance (Hirano et al., 2016), which points to different waves of gene expression in different late memory phases.

The reported upregulation of gene expression in MB neurons after learning, although tremendously informative in pursuing which genes are involved with the persistence of memories, does not imply that protein synthesis is necessary in these neurons for LTM.

Different efforts have been made to interfere with protein synthesis in MB specific neurons in the search of a causative effect, and the results have been controversial in the context of aversive associative memory (Yu et al., 2006; Chen et al., 2012; Hirano et al., 2013). This is probably due to the different drivers utilized and the different efficiencies of the genetic approaches selected. The use of gene editing to generate a CrebB conditional knock-out allele allowed to determine that CrebB function is necessary in KC-α/β and KC-α′/β′ neurons for appetitive LTM (Widmer et al., 2018b). These results nicely correlate with the known requirement of synaptic output in the same groups of neurons for LTM (Trannoy et al., 2011; Cervantes-Sandoval et al., 2013). On the contrast, CrebB function is not necessary in γ neurons, which synaptic output is required for STM expression, but not for LTM aversive or appetitive memory (Cervantes-Sandoval et al., 2013). It is unlikely that appetitive and aversive memories generate such a strikingly different genetic program regarding the requirement of CrebB in KC, and the evidence points in the direction of a general requirement of CrebB-dependent gene expression in the MB.

Similar experiments have been performed to identity translational control mechanisms required for LTM and cells in which these mechanisms are required *in vivo*. For instance, the fragile X protein (FMRP), an RNA binding protein, is the most frequent cause of intellectual disability in males. FMRP is a known translational regulator involved, for instance, in the siRNA pathway and mGluR-mediated translation control. FMRP was first associated with LTM formation due to its interaction with *staufen* (Dubnau et al., 2003), components of the RNAi pathway (Jin et al., 2004) and a RNP granule complex containing neuronal translational components (Sudhakaran et al., 2014). *Drosophila* FMRP mutants show specific defects in LTM and inhibiting FMR, using a RNAi line in KC selectively, blocks LTM (Bolduc et al., 2008). Interestingly it was also shown that LTM defects in FRMP mutants are ameliorated by protein synthesis inhibition (Bolduc et al., 2008).

Long term memory-induced differential gene expression has been reported in other cell types outside of KC, like DAL (Chen et al., 2012), MBONα2, MBONα3 (Crocker et al., 2016), and glia (Matsuno et al., 2015). These results correspond with a known requirement of CrebB activity in DAL (Chen et al., 2012; Hirano et al., 2013), MBONα3 and others (Wu et al., 2017) Table 2 summarizes the neuronal subtypes in which translation has been shown to be specifically required for different types of LTM formation. The table contains also long-term olfactory habituation, a type of non-associative memory which, unlike associative memory, involves plasticity of GABAergic synapses onto PN in the antennal lobe (Das et al., 2011; McCann et al., 2011; Sudhakaran et al., 2014).

**TABLE 2:** Neuronal subtypes in which translation has been shown to be specifically required for different types of long-term memory.

**Neuronal population**	**Where**	**Gal-4 line**	**Protein**	**Protein role**	**Type of inhibition**	**References**	**Memory Paradigm**
KC	KC α/β	c709	CREB	Transcription factor	*dCreb2-b* repressor and *Creb* flippase recombinase	Krashes and Waddell, 2008; Widmer et al., 2018b	Appetitive conditioning
	KC α/βα′/β′	c722	CREB	Transcription factor	*dCreb2-b* repressor	Krashes and Waddell, 2008	Appetitive conditioning
	KC α/β; γ	MB247	CREB	Transcription factor	*dCreb2-b* repressor	Krashes and Waddell, 2008	Appetitive conditioning
	KC all	OK107	CREB	Transcription factor	*Creb* flippase recombinase	Widmer et al., 2018b	Appetitive conditioning
	KC α′/β′	c305a	CREB	Transcription factor	*Creb* flippase recombinase	Widmer et al., 2018b	Appetitive conditioning
MBON	MBON α3	G0239	CREB	Transcription factor	*Creb* flippase recombinase	Widmer et al., 2018b	Appetitive conditioning
KC	KC all	OK107	FMRP	RNA binding protein	Fmr RNAi	Bolduc et al., 2008	Aversive conditioning
KC	KC α/β; γ	MB247-Switch	CREB	Transcription factor	*dCreb2-b* repressor	Hirano et al., 2013	Aversive conditioning
Projection Neurons	PN	GH146			CaMKII 3′UTR EYFP reporter	Ashraf et al., 2006	Aversive Conditioning
DAL	DAL	E0946, G0338, G0431			Translation (by targeted expression of RICIN(ts)	Chen et al., 2012	Aversive Conditioning
MBON	MBON-α3	E0067, E1132, G0239, MB082C			Translation (by targeted expression of RICIN(ts)	Pai et al., 2013; Wu et al., 2017	Aversive Conditioning
MBON	MBON-γ3,γ3β′1	VT16811, VT48852			Translation (by targeted expression of RICIN(ts)	Wu et al., 2017	Aversive Conditioning
MBON	MBON-β′2mp	VT41043, VT44170			Translation [by targeted expression of RICIN(ts)]	Wu et al., 2017	Aversive Conditioning
KC	KC	fru	Orb2	Prion-like protein–translational repressor/activator	Rescue using UAS-orb2	Keleman et al., 2007	Courtship conditioning
	KC α/β; α′/β′	c722	Orb2	Prion-like protein–translational repressor/activator	Rescue using UAS-orb2	Keleman et al., 2007	Courtship conditioning
	KC α/β; γ	MB247	Orb2	Prion-like protein–translational repressor/activator	Rescue using UAS-orb2	Keleman et al., 2007	Courtship conditioning
Local interneurons	LN	LN1	Ataxin-2	RNA-binding protein	Atx2 RNAi	Das et al., 2011	Olfactory habituation
	LN	LN1	FMRP	RNA-binding protein	dFMR1 RNAi	Sudhakaran et al., 2014	Olfactory Habituation
	LN	GAD1	Ataxin-2	RNA-binding protein	Atx2 RNAi	Das et al., 2011	Olfactory habituation
Projection neurons	PN	GH146	Ataxin-2	RNA-binding protein	Atx2 RNAi	McCann et al., 2011	Olfactory habituation
	PN	GH146	FMRP	RNA binding protein	dFMR1 RNAi	Sudhakaran et al., 2014	Olfactory habituation
	PN	VPN	Ataxin-2	RNA-binding protein	Atx2 RNAi	McCann et al., 2011	Olfactory habituation

While we have come a long way since the days of forward behavioral screenings that detected what we now consider classic memory genes, there is still much to decipher regarding the temporal and spatial requirements of gene expression in LTM. We know now that alternative splicing variants can be differentially expressed due to memory acquisition (Petruccelli et al., 2020), and we know that engram cells are not homogeneous regarding their transcriptional profiles (Shih et al., 2019). These features could lead to disregard of important genetic effects when different cells are pooled together. Moreover, different subcellular localizations might undertake protein synthesis independently to allow selected synapses to be regulated.

## Prion-Like Proteins and Ribonucleoprotein Granules

So far, we have reviewed how the formation of LTM requires the synthesis of plasticity-related proteins (Prp). We have discussed that the formation of LTM requires the initiation of a nuclear program that will maintain the induced modifications in specific, relevant synapses, which in turn will maintain those memories, or behavioral modifications, for long periods, sometimes for the lifetime of the animal. These findings posit two substantial problems that the brain needs to resolve: First, a single neuron contains on average hundreds to thousands of synaptic inputs and outputs. For example, in *Drosophila*, a typical KC-α/β neuron has approximately 500 inputs and 200 output connections with different neuronal types including, APL, dorsal-paired medial neuron (DPM), DAN, MBON and other KCs (Li et al., 2020). If the nuclear program initiated by encoding LTM affected all synapses equally, it would significantly decrease the computing volume of a neuron. The cell biology of neurons requires the existence of a mechanism that allows them to specifically capture global changes (activation of a nuclear program) and constrain the plasticity-related modifications to specific, relevant synapses.

Second, if the synaptic changes that encode a particular long-lasting memory are formed and maintained by the synthesis of new proteins, how can the lifetime of these memories surpass the lifetime of these biomolecules that sustain them? Once more, neurons need a cellular mechanism to self-perpetuate changes induced by plasticity.

The need for nuclear gene expression in long-term plasticity led to a key question: how can a genomic change lead to long-term modifications in specific relevant synapses and not others? Evidence that synapse specific long-term plasticity exists and can occur by interaction of a local activity-dependent synaptic mark/tag that then allows protein targeting to or “capture” by that synapse was demonstrated and developed by Frey and Morris into what is now known as the synaptic tagging and capture hypothesis (Goelet et al., 1986; Frey and Morris, 1997; Ballarini et al., 2009; Redondo and Morris, 2011; Moncada et al., 2015). Martin and Kandel provided evidence for such a synaptic tag with single-neuron precision using a reduced *in vitro* preparation. They grew an *Aplysia* sensory neuron with a branched axon forming synapses with two separated motor neurons. As expected, when a single pulse of serotonin was applied to one synapse, short-term facilitation (STF) was induced in this branch and no the other. On the other hand, five pulses of serotonin-induced protein synthesis-dependent long-term facilitation (PSD-LTF), again only in the stimulated synapse. This indicated that the effects of the nuclear program somehow were restricted to the relevant synapse. The key finding came when a single pulse of serotonin to one branch induced PSD-LTF when paired with five pulses applied to the other branch. This indicated that a single serotonin pulse is sufficient to generate a mark or “tag” at that synapse that then can “capture” Prp produced by the nuclear program initiated by the five serotonin pulses on the other branch (Martin et al., 1997). These results complemented the aforementioned observations from Frey and Morris (1997), and were later followed by other remarkable studies, like the one carried out by Barco and colleagues on the synaptic capture of long-term potentiation (LTP) in slices of mammalian hippocampus (Barco et al., 2002).

Nevertheless, an important question remains unanswered: how are Prp delivered to specific synapses or “capture” by these “tags?”. We can imagine three different possibilities. (1) Newly transcribed mRNAs are translated in the somatic cytoplasm and proteins transported to and captured by the relevant synapses; (2) newly transcribed mRNAs are transported to relevant synapses, where translation takes place; and (3) mRNAs encoding Prp are constitutively present and stored at synapses in a silenced state, where they are later translated in response to synaptic activity. In this case, mRNAs may be located at synapses either as individual messenger ribonucleoproteins (mRNPs) or as mRNP assemblies–RNA granules.

The first scenario implies that mRNA translation occurs mainly in the soma, and the proteins required for synaptic plasticity are specifically transported to active “tagged” synapses (Frey and Morris, 1997). This idea fits within the framework of “synaptic-tagging.” Nevertheless, several arguments against this possibility come to mind: First, there are intrinsic temporal limitations: newly synthesized protein transport to tagged-synapses is necessarily limited by the rate of intracellular diffusion and/or active transport mechanisms. Second, it is an inefficient strategy because only a small fraction of synthesized protein will reach the destination where it is required (the excess would presumably be turned over). And last, many studies have directly shown that mRNAs are translated locally at active synapses (Martin et al., 1997; Aakalu et al., 2001) and that this local mRNA translation is required for long-term synaptic plasticity.

In the second scenario, mRNAs could be transported from the soma down the axons and dendrites where they are selectively translated. Several lines of evidence support this model: not only has *in situ* hybridization shown that several mRNA are enriched in dendrites and axons, but also mRNAs are known to be present on granules that are transported by kinesin motors to distal segments of neurites (Garner et al., 1988; Burgin et al., 1990; Bassell et al., 1998). Moreover, recently RNA sequencing combined with *in situ* hybridization and Nanostring, a technique which allows high resolution visualization of single mRNA molecules and permits to obtain quantitative estimates of the mRNA abundance (Geiss et al., 2008), have shown that >2,500 mRNAs are localized and translated in axons and dendrites of pyramidal neurons in mice (Cajigas et al., 2012; Holt et al., 2019). In addition, mRNA are known cargos of motor proteins for axonal transport (Lee, 2016).

The third mechanism to explain local protein synthesis requirement for synapse-specific plasticity posits that subsets of mRNAs encoding plasticity factors are stored in a repressed state at or near synapses; their translation can be derepressed and/or activated by local synaptic signaling. In support of this model, local translation has clearly been shown to occur in dendrites (Ashraf et al., 2006), and in dendrites surgically disconnected from soma (Kang and Schuman, 1996; Martin et al., 1997; Aakalu et al., 2001). Additionally, activity-induced *de novo* translation of dendritic mRNAs, including CaMKII, has been shown to occur at active synapses *in vivo* in both mice and flies (Miller et al., 2002; Ashraf et al., 2006). Moreover, the 3′UTR segment of CaMKII allows the mRNA to specifically localize to dendrites, and mutants lacking this segment show altered memory and synaptic plasticity (Miller et al., 2002; Ashraf et al., 2006). These data are consistent with activity-induced synaptic translation of dendritically localized mRNA being essential for long-term synaptic plasticity *in vivo*.

Considering that mRNAs are synthesized in the cell body and that there is evidence that local translation is needed, it appears most likely that mRNAs are not translated until they reach the relevant synapses, where they become translationally active. In 2003 Si et al. (2003), reported in *Aplysia* that a neuron-specific isoform of the cytoplasmic polyadenylation element-binding protein (CPEB) regulates plasticity-related synaptic protein synthesis in an activity-dependent manner. They found that *Aplysia* CPEB is upregulated locally in synapses when these are stimulated by a single pulse of serotonin. This upregulation, which occurs at translational level, is required to maintain PSD-LTF and structural changes associated with it, but it is dispensable to form STF (Si et al., 2003; Miniaci et al., 2008). A single serotonin pulse could induce LTF in cells with no cell bodies, which indicated that CPEB induction did not require a message from the nucleus.

The idea that prion-like proteins could provide a self-sustaining mechanism for memory maintenance was hypothesized in the late 90s and supported by direct studies in *Aplysia* and *Drosophila* (Tompa and Friedrich, 1998; Roberson and David Sweatt, 1999; Si et al., 2003). Si et al. (2010) showed that *Aplysia* CPEB has prion-like properties and can have two distinct functional states. As other prions, the prion-like conformation forms oligomers or aggregates, it is self-perpetuating, and it was found to stimulate translation. Additionally, CPEB lacking the 252 N-terminal, that contains the prion-like domain, fails to form these aggregates, but surprisingly it can still form aggregates if expressed along full-length CPEB. This means that the N-terminal is necessary for aggregation initiation but is dispensable for recruitment to an existing oligomer. Finally, using thioflavin labeling to visualize β-sheets assemblies and document filamentous CPEB structures under electron microscopy, the authors showed that CPEB multimers are amyloids (Si et al., 2010).

One of two *Drosophila* homologs of CPEB is called Orb2. Similar to *Aplysia* CPEB, Orb2 is also required for the formation of PSD-LTM after courtship and olfactory conditioning (Keleman et al., 2007; Krüttner et al., 2012, 2015; Majumdar et al., 2012; White-Grindley et al., 2014; Li et al., 2016; Gill et al., 2017). Like the neuronal *Aplysia* CPEB, Orb2 can be found in monomeric and in amyloid-like oligomeric forms. Interestingly, neuronal stimulation of KC’s using thermogenetics or neurotransmitter feeding induces Orb2 oligomerization and these aggregates are enriched in synaptic membranes (Majumdar et al., 2012). Moreover, using a proteomics approach, Orb2 was found to interact with proteins that fall into three categories: synaptic proteins (i.e., Snap25, Syt7, and Dlg), mRNA binding proteins (i.e., Pabp2, and Pof), and translation initiation proteins (eIF4E and eIF33-p40) (White-Grindley et al., 2014). Orb2 isoform Orb2A is critical for oligomerization, and a single point mutation (F5Y) selectively affects its aggregation properties as well as courtship conditioning and appetitive olfactory conditioning late-LTM (Majumdar et al., 2012). Additional proteins also contribute to the aggregation of Orb2 and its oligomer stability. Transducer of Erb2 (Tob), a known regulator of cellular growth, was found to interact with Orb2 and facilitate and stabilize oligomers’ formation after neuronal activity (White-Grindley et al., 2014). Importantly, other proteins, like CG13928, interact exclusively with the monomers and seem to help with the translational repression function (Khan et al., 2015). It is essential to mention that Orb2A monomers and oligomers bind equally to 3′UTR of target genes. This means that Orb2A binding properties to mRNA are not affected by its conformational changes. In a study using purified monomeric or oligomeric Orb2A and Orb2B in an *in vitro* translation assay, Si and colleagues made the remarkable finding that while the monomeric conformation is a translational repressor, oligomeric Orb2A or Orb2B, together with protein partners like CG4612, act as translational activators (Khan et al., 2015).

A more recent study has elucidated the atomic structure of Orb2A oligomers using CryoEM microscopy. Orb2 forms threefold-symmetric amyloid filaments of 75 nm in length. Once again, these filaments’ formation transformed Orb2 from a translation repressor to an activator and an initiator for further aggregation (Hervas et al., 2020). These studies provide evidence that prion-like proteins like *Drosophila* Orb2 could function as a molecular tag during LTM formation.

But how memory encoding events regulate Orb2? Previous studies had shown that Orb2 expression is induced by neuronal activity. We have previously mentioned that Orb2 was detected in transcriptomic analysis as differentially expressed due to memory acquisition (Jones et al., 2018). Moreover, it was recently reported that Orb2A mRNA is expressed as a non-translatable, unspliced mRNA in the adult brain. Notably, only long-term plasticity-inducing activity, but no other neuronal activity, increases the mature spliced mRNA and therefore Orb2A translation. The splicing regulator Pasilla controls the abundance of spliced mRNA levels (Gill et al., 2017).

**FIGURE 2:**
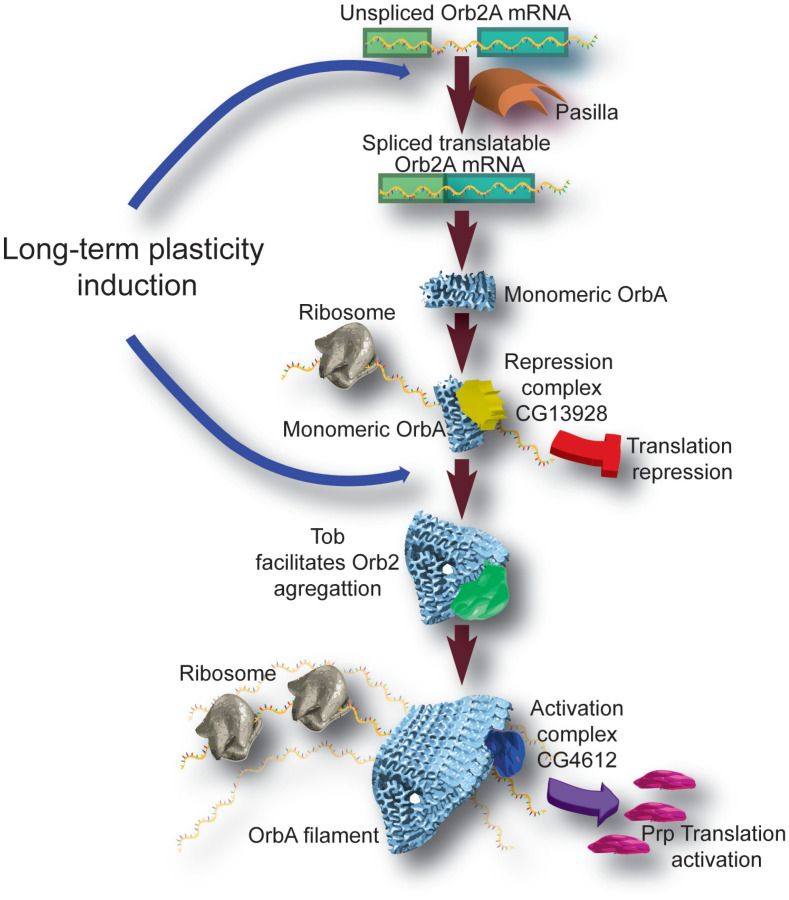
Schematic of role and mechanism of prion-like protein Orb2 in the formation of long-term memory. Long-term inducing plasticity, like appetitive olfactory conditioning engages the splicing regulator Pasilla. Orb2A mRNA is initially transcribed as an unspliced untranslatable mRNA in neuronal cells. Activated Pasilla controls the production of protein-coding translatable spliced Orb2 mRNA. Before oligomerization, CG13928 binds Orb2 monomer and recruits the translation repression complex. In this state Orb2A functions as a translational repressor. Synaptic activation leads to additional Orb2 synthesis. Orb2 is bound and stabilized by the transducer of ERBB2, Tob. This binding promotes Orb2 oligomerization. CG4612 binds aggregates Orb2 and recruits a translation promoting complex. In this state Orb2 works as a translational activator. Orb2 protein switches from repressing to activating translation when aggregates and forms amyloid-like oligomers. It is proposed that the translation activation of Orb2 is involved in the translation of plasticity-related proteins like tequila, PKC, and Murashka and therefore sustains long-term memory. Because prion-like proteins are self-assembling molecules and can self-perpetuate, Orb2 function provides an attractive mechanism for synapse-specific plasticity and memories that outlast the lifetime of individual protein molecules.

Altogether these studies provide at least seven lines of evidence that prion-like proteins like *Drosophila* Orb2 could function as a molecular tag of the relevant synapses during synaptic plasticity: A, Orb2A is enriched in synaptic membranes and interacts with multiple synaptic proteins. B, Orb2 is upregulated in an activity-dependent manner. C, this activation is restricted to the stimulated (relevant) synapse. D, Orb2A activation undergoes a change from monomeric to oligomeric form. E, the monomeric form, which could presumably be present in most synapses, functions as a translational repressor, therefore inhibiting plasticity-related protein translation. Upon activation, Orb2A aggregates and becomes a translational activator, which initiates plasticity-related protein expression in the relevant synapses. F, Orb2 is involved in the translational regulation of proteins previously showed to be necessary for LTM, like Tequila, PKC, and Murashka (Mastushita-Sakai et al., 2010; Stepien et al., 2016). Finally, prion-like proteins are self-assembling molecules and can self-perpetuate. While there is not yet incontrovertible proof that all of these features of Orb2 are relevant *in vivo*, these studies provide an attractive mechanism for synapse-specific plasticity and memories that outlast the lifetime of individual protein molecules (Figure 2).

The proposed ability of Orb2 oligomers to act as translational activators *in vivo* contrasts with other prion-like proteins such as yeast Rim4, also involved in translational control (Berchowitz et al., 2015). The amyloid-like form of Rim4 is actively regulated and represses translation, while clearing of amyloids releases mRNA into a translationally active pool.

Another function of prion-like domains on RNA-binding proteins takes place in membrane-less organelles called mRNP granules. Many lines of evidence argue that mRNAs are transported to and localized in neural processes through mRNPs (Knowles et al., 1996; Krichevsky and Kosik, 2001; Kanai et al., 2004; Kiebler and Bassell, 2006). Additional observations, consistent with studies on Rim4, show that these granules can be disassembled in response to synaptic activity, freeing previously sequestered and repressed mRNAs for translation (Zeitelhofer et al., 2008). Moreover, recent studies in *Drosophila* show that mutations in the prion-domain related element of Ataxin-2 specifically disrupt mRNP granule formation while also causing specific defects in long-term behavioral habituation (Bakthavachalu et al., 2018). These observations support: (a) a role for at least some prion-like domains in RNP granule formation and (b) provide empirical support for the “sushi-belt” model, which posits that single mRNP granules in dendrites “service” multiple synapses, thereby providing increased temporal and energetic efficiency to local translational events that underlie long-term plasticity (Doyle and Kiebler, 2011; Williams et al., 2016).

It is important to underline that all three models mentioned here have some shortcomings and cannot be excluded completely. It is possible that different mechanisms are used under different contexts. In addition, there is a possibility that they could co-exist.

## Concluding Remarks

Considerable progress has been made in understanding how experiences trigger a nuclear program of gene expression and how this results in long lasting changes in behavior. New whole-genome transcriptomic studies identified several proteins regulated by this nuclear program, and we are starting to learn how their effects are restricted to the specific and relevant nodes of the neural circuit that sustain a particular LTM.

Nevertheless, some critical questions remain unsolved: first, new whole transcriptomic analyses have helped identifying many possible players involved in LTP formation. Some of these genes are long-known regulators of long-term synaptic plasticity, and their mechanism of action has been described. However, the role of many other candidates is completely unknown. Considerable efforts will be needed to gather mechanistic insight on the molecular function of independent genes, and bring them together in a coherent sequence that explains LTM.

Another main remaining question refers to the temporal requirement of transcription and translation for the formation, consolidation, and maintenance of LTM. Studies across species, from insects and mollusks to mammals, have shown not only that new gene expression and local protein synthesis is broadly required for LTM formation, but also that protein synthesis is required during multiple phases of LTM, acquisition (learning), consolidation, reconsolidation and, potentially, maintenance; opposed to the classic theory which poses protein synthesis as required just for memory consolidation.

In *Aplysia*, blocking synaptic translation with PSI 24 and 48 h after training impairs LTM expression. Surprisingly, if those PSI are given 72 h after training, this memory impairment is no longer observed (Miniaci et al., 2008). These results suggest that translation is required after training during a specific time window, when memories are labile and can be disrupted, but once the changes induced by LTM formation are stabilized, protein synthesis is no longer required for its maintenance. Similarly, partial training could restore LTM in *Aplysia*, which alone is insufficient to induce LTM, after protein synthesis inhibitor-induced amnesia (Pearce et al., 2017). This memory restoration can only occur if the protein synthesis inhibitor is injected after training (Pearce et al., 2017). These results are also in accordance with a “classic” paper published by Squire and Barondes (1972), in which mice injected with PSI after training couldn’t retrieve 24 and 48 h memory but had normal 72 h memory (Squire and Barondes, 1972). Interestingly, Pearce *et al.*, 2017 went one step further, pointing at epigenetic changes, such as DNA methylation, as base for LTM consolidation mechanism (Pearce et al., 2017).

Reflecting the increasing complexity of LTM, there are plenty of examples in *Drosophila* where either cellular or genetic insults, or even only particular behavioral experiences, can form long-lasting memory that are completely protein-synthesis independent, like anesthesia-resistance memory (Shuai et al., 2010; Berry et al., 2012; Cervantes-Sandoval et al., 2016; Zhang et al., 2016; Zhao et al., 2019; Eschment et al., 2020). Inhibition of the small G protein Rac1 or knockdown of the scaffolding protein Scribble disrupt normal forgetting and result in robust long-lasting protein synthesis independent memories (Shuai et al., 2010; Cervantes-Sandoval et al., 2016). Similarly, single-trial contextual olfactory aversive conditioning induces the formation of protein-synthesis independent long-lasting memory (Zhao et al., 2019). If, in these examples, long-lasting memories are protein-synthesis independent, how are the synaptic modifications that resulted in the formation of these memories maintained?

The mysterious question of how memories last a lifetime remains open.

## Author Contributions

CR, MR, TB, and IC-S wrote parts of the manuscript. TB and IC-S edited the final version of the review. All authors contributed to the article and approved the submitted version.

## Conflict of Interest

The authors declare that the research was conducted in the absence of any commercial or financial relationships that could be construed as a potential conflict of interest.
